# AGING STRONG 2020: INTERVENTIONS TO IMPROVE LONELINESS AMONG OLDER ADULTS

**DOI:** 10.1093/geroni/igz038.657

**Published:** 2019-11-08

**Authors:** Charlotte Yeh, Daniel Russell, James Schaeffer

**Affiliations:** 1 AARP Services, Inc., Washington, District of Columbia, United States; 2 Iowa State University, Ames, Iowa, United States; 3 Optum, Ann Arbor, Michigan, United States

## Abstract

Research confirms serious and concerning health implications for lonely and socially isolated older adults. Studies consistently demonstrate that older adults who are lonely or socially isolated have higher rates of depression, more health conditions, and greater mortality. AARP Services, Inc. (ASI) and UnitedHealthcare (UHC) are committed to the health and well-being of insureds in AARP® Medicare Supplement Plans insured by UnitedHealthcare Insurance Company (for New York certificate holders, UnitedHealthcare of New York), recognizing that health and wellness should be promoted on a holistic level to ensure successful aging. As part of this commitment, a research initiative entitled Aging Strong 2020 has been developed. Its purpose is to impact insureds’ personal and social investments in their well-being Thus a related series of interventions are aiming to increase resilience by focusing on enhanced purpose in life, social connectedness, and optimism. This symposium will specifically discuss these efforts related to social connectedness and how they have improved well-being among lonely older adults. First discussed is the prevalence and outcomes of loneliness in a large national survey. Interventions include use of animatronic pets, a telephonic reminiscent memory program, and an online self-compassion mindfulness program. Findings from these initiatives demonstrate that interventions designed to improve loneliness and well-being among lonely older adults can contribute to the holistic model of health.

